# The Menopause Rating Scale (MRS) as outcome measure for hormone treatment? A validation study

**DOI:** 10.1186/1477-7525-2-67

**Published:** 2004-11-22

**Authors:** Lothar AJ Heinemann, Thai DoMinh, Frank Strelow, Silvia Gerbsch, Jörg Schnitker, Hermann PG Schneider

**Affiliations:** 1 Center for Epidemiology & Health Research Berlin, Invalidenstr. 115, 10115 Berlin, German; 2 Schering Deutschland GmbH, Max-Dohrn-Str. 10, 10589 Berlin, Germany; 3 Institut für Angewandte Statistik, Artur-Ladebeck-Str.155, 33647 Bielefeld, Germany; 4 University Muenster, Dept. Obstetrics and Gynecology, Von-Esmarch-Strasse 56, 48149 Muenster, Germany

**Keywords:** MRS, Menopause, Hormone treatment, Validity, health-related Quality of Life, Questionnaire

## Abstract

**Background:**

The Menopause Rating Scale is a health-related Quality of Life scale developed in the early 1990s and step-by-step validated since then. No methodologically detailed work on the utility of the scale to assess health-related changes after treatment was published before.

**Method:**

We analysed an open, uncontrolled post-marketing study with over 9000 women with pre- and post-treatment data of the MRS scale to critically evaluate the capacity of the scale to measure the health-related effects of hormone treatment independent from the severity of complaints at baseline.

**Results:**

The improvement of complaints during treatment relative to the baseline score was 36% in average. Patients with little/no complaints before therapy improved by 11%, those with mild complaints at entry by 32%, with moderate by 44%, and with severe symptoms by 55% – compared with the baseline score. We showed that the distribution of complaints in women before therapy returned to norm values after 6 months of hormone treatment. We also provided weak evidence that the MRS results may well predict the assessment of the treating physician. Limitations of the study, however, may have lead to overestimating the utility of the MRS scale as outcome measure.

**Conclusion:**

The MRS scale showed some evidence for its ability to measure treatment effects on quality of life across the full range of severity of complaints in aging women. This however needs confirmation in other and better-designed clinical/outcome studies.

## Background

The Menopause Rating Scale (MRS) was initially developed in the early 1990s [1,2] to measure the severity of age-/menopause-related complaints by rating a profile of symptoms.

The validation of the MRS began some years ago [2-6] with the objectives (1) to enable comparisons of the symptoms of aging between groups of women under different conditions, (2) to compare severity of symptoms over time, and (3) to measure changes pre- and post-treatment [4-6].

Development and standardization of the scale were published elsewhere [2]. In brief, the standardization of this scale was performed on the basis of a representative sample of 500 German women aged 45–60 years in 1996. A factorial analysis was applied to establish the raw scale of complaints or symptoms. Statistical methods were used to identify the dimensions of the scale. Finally, three dimensions of symptoms/complaints were identified: a psychological, a somatic-vegetative, and a urogenital factor that explained 59 % of the total variance [2]. This is indicative for a high efficiency of a scale with only 11 items – compared to other international scales. Reference values for the severity of symptoms or complaints were calculated based on a population sample [2]. The scale consisting of 11 items is self-completed by the woman. A 5-point rating scale permits to describe the perceived severity of complaints of each item (severity 0 [no complaints]...4 scoring points [very severe symptoms]) by checking the appropriate box. The composite scores for each of the dimensions (sub-scales) are based on adding up the scores of the items of the respective dimensions. The composite score (total score) is the sum of the dimension scores. Details as how to apply and evaluate the scale were published [8,9] and can be also obtained from the website .

The scale was defined as a menopause-specifc, health-related quality of life scale (HRQoL), because the profil of complaints in this scale importantly determines the HRQoL of women in this age span. Moreover, a good correlation between the results obtained with the MRS scale and the generic QoL scale was observed [6].

The MRS scale became internationally well accepted as far as the usage many countries is concerned. The first translation was into English [7]. Other translations followed [8], i.e. taking international methodological recommendations [10-12] into consideration. Currently, the following versions are available: Brazilian, English, French, German, Indonesian, Italian, Mexican/Argentine, Spanish, Swedish, and Turkish language. These versions are available in a published form, and can be downloaded in PDF-format from the internet (see reference 8 and ).

Like in other health-related QoL scales, it is a challenge to satisfy the demands of a clinical utility and outcomes sensitivity. A comprehensive overview regarding conventional psychometric requirements of test reliability and validity were recently published elsewhere [9]. It is the aim of this paper is to share methodological information about the capacity of the scale to assess changes after hormone treatment since no methodologically detailed work in this regard was published before.

## Methods

A multicenter, open post-marketing study was conducted with a product for hormone therapy (CLIMEN^® ^= 2 mg estradiol valerate/2 mg estradiol valerate + 1 mg cyproterone acetate) using the MRS scale as outcome measure under routine conditions of office-based gynaecologists. The study was described in detail elsewhere [6].

In brief, 1801 gynaecologists from all parts of Germany participated in the study on a voluntary basis. 10,904 women who required hormone treatment were included. The median age was 49 years. Beside others, the MRS scale was documented before therapy and 6 months after starting the hormone treatment.

A specific problem was the transformation of an older MRS version into the advanced, relatively broad validated current version of MRS. The old version of the MRS was read by the physician and the patient answered to which extend she perceived suffering from a specific symptom, and if yes to which extend. The new scale is self-completed by the woman without interaction with the physician. The symptoms itself are the same in both scales. Nevertheless, this is a methodological limitation. We, however, are not interested in the absolute score values but relative changes after treatment compared to before. In addition, the scoring system of the old version was adjusted into the new coding system using a linear transformation. Additionally, one question of the old version was split into two questions – as recommended for the current version of the MRS (see later discussion on limitations of the study).

The statistical analyses were performed with the commercial statistical package SAS 8.2.

## Results

Altogether, data of 9311 women were available for most of our analysis. However, the sample size varied slightly depending on the variables used because we had also missing information in a few variables.

The mean age was 49.8 years (SD 6.4). About half of the participating women were still perimenopausal (51.9%) or were already in the postmenopausal phase (48.1%). The mean body mass index was not eye-catching with 24.7 (SD 3.7).

The improvement of the health-related quality of life (HRQoL) – measured with the MRS scale – is described in Table 1. The means and SD of the scoring points of the total scale (and the three subscales) can be seen at baseline (before therapy) and after six months of hormone treatment. Significant declines of the mean scores were observed after treatment indicating an improvement of HRQoL altogether and in the three subscales of the MRS.

**Table 1:** Means (SD) of MRS scores at baseline (before therapy) and at end of observation (after therapy. Improvement of scores after therapy by absolute difference in scoring points. ^§^Total scale and for each subscale.

	**n**	Scores before	Scores after	Absolute change	Percent (%) change	P**
**Total scale**	9311	11.0 (8.2)	1.7 (3.2)	9.3 (7.4)	36.1 (20.6)	<0.0001
**Psychological subscale**	9311	4.5 (4.1)	0.7 (1.7)	3.8 (3.7)	34.5 (27.1)	<0.0001
**Somatic subscale**	9311	4.2 (3.2)	0.5 (1.2)	3.6 (3.0)	37.3 (23.1)	<0.0001
**Urogenital subscale**	9311	2.3 (2.6)	0.5 (1.1)	1.8 (2.3)	24.5 (25.3)	<0.0001

^§ ^Summary score "before therapy" minus "after therapy"

* Percent (%) change compared with the change before treatment: pre-treatment score minus post-treatment divided by pre-treatment score multiplied by 100 (%)

** Paired t-test for dependent samples: significance of the absolute difference

Apart from the comparison of means, we calculated the relative improvement compared with the situation before therapy (baseline) to better understand the magnitude of change after therapy (Table 1), i.e. in absolute and relative terms. There was not much difference in relative improvement (%) among subscales (all highly significant): In average, the scores improved by one third after six months hormone treatment.

The scale is able to measure an improvement in patients starting with "no/little complaints" (total score = 0–4), "mild" (5–8), "moderate"(9–15), and "severe" (16 + points) before therapy (= baseline). This is presented in Table 2: The more severe the complaints were before treatment the better the effect regarding relative improvement of symptoms measured by the MRS, which gives evidence for the clinical utility of the MRS as outcome measure.

**Table 2:** Relative change of MRS scoring points as percent of the baseline total score: Mean values (SD) of the relative change (% improvement of the complaints) in four categories of severity at baseline.

	Severity of complaints at baseline			
	**No/little (0–4)**	**Mild (5–8)**	**Moderate (9–15)**	**Severe (16+)**
	Means (SD) (n = 2460)	Means (SD) (n = 1693)	Means (SD) (n = 2592)	Means (SD) (n = 2566)

Total score	10.8 (10.6)	32.2 (9.8)	43.9 (11.8)	55.1 (13.8)
Psychological score	6.0 (14.7)	27.6 (21.5)	43.7 (20.6)	57.1 (17.9)
Somatic score	13.8 (17.3)	34.4 (18.5)	44.1 (16.9)	54.8 (15.9)
Urogenital score	5.7 (13.9)	17.0 (20.6)	27.5 (23.6)	44.4 (22.6)

It is interesting to compare the HRQoL before and after hormone treatment with the norm values of MRS obtained in an average population of aging women, i.e. not patients as in our post-marketing study. To this end, we compared only the MRS total scores in patients with the average female population (Table 3). It became evident in this crude and simple comparison, that the severely deteriorated distribution of complaints in the patient group before therapy – compared with the normal population – improved after therapy remarkable, i.e., at least as far as the total score of the MRS is concerned. The three subscales showed a similar tendency towards the better. The extremely high proportion of patients without complaints immediately after therapy could be due to a selection problem in this post-marketing study and the application of the physician-administered version of the MRS (see discussion).

**Table 3:** Level of complaints in the population in percent (%) compared with patients of the Climen treatment study: Frequency distribution before/after therapy compared with population norm values*

		Frequency in *patients*: Percent (%)in four categories of severity	
Severity of complaints	**Population % Standard**	Before therapy %	After therapy %
Total score			
No or little (-4)	**48**	**26.4**	**86.8**
Mild (5–8)	**25**	**18.2**	**8.4**
Moderate (9–15)	**20**	**27.8**	**4.0**
Severe (16+)	**8**	**27.6**	**0.8**
Psychological score			
No or little (-1)	**48**	**30.7**	**82.5**
Mild (2–3)	**23**	**17.0**	**10.4**
Moderate (4–6)	**20**	**22.2**	**5.5**
Severe (7+)	**9**	**30.1**	**1.6**
Somatic score			
No or little (-2)	**53**	**35.7**	**93.0**
Mild (3–4)	**24**	**22.7**	**5.1**
Moderate (5–7)	**15**	**25.5**	**1.6**
Severe (8+)	**8**	**16.1**	**0.3**
Sexual score			
No or little (0)	**64**	**37.3**	**76.8**
Mild (1)	**13**	**13.3**	**11.6**
Moderate (2–3)	**16**	**22.2**	**8.7**
Severe (4+)	**7**	**27.3**	**2.9**

* The population data came from the standardization of the MRS scale [2,3]

The treating gynaecologist (who also applied the MRS scale) assessed individually the efficiency of the hormone treatment in the above mentioned intervention study. The gynaecologist's expert opinion regarding treatment efficiency was categorized into two categories for the purpose of this analysis: *successful *(very effective and effective) and *not successful *(little, no, or negative effects). This alternative variable was then used for the comparison with the alternative "success-variable" based on MRS (total score only): "*successful*" (5 and more points reduction after therapy compared with baseline test) and "*not successful*" (less than 5 scoring points reduction after therapy compared with baseline test).

The prediction of the expert opinion of the treating gynaecologist with the MRS data seems to be good: sensitivity (correct prediction of a positive assessment by the physician) 70.8% and specificity (correct prediction of a negative assessment by the physician) 73.5% (Table 4).

**Table 4:** Prediction of a positive assessment by the physician concerning "successful treatment" by means of the MRS scale (total score)."Not successful" was defined for the MRS as: less than 5 scoring points improvement at the end of the HRT treatment compared with "before treatment".

	MRS: not successful	MRS: successful	Total
Doctor: not successful	311	112	423
Doctor: successful	2570	6227	8797
Total	2881	6339	9220

Sensitivity: 70.8%

Specificity: 73.5

## Discussion

The MRS scale was developed (a) to assess symptoms of aging/menopause (independent from those that are disease-related) or HRQoL between groups of women under different conditions, (b) to evaluate the severity of symptoms over time, and (c) to measure changes pre- and post hormone replacement therapy. The aim of this paper was to empirically demonstrate that the latter claim is evident.

Reliability and validity are important to show the usefulness of the scale as a clinical utility in monitoring treatment effects – once all other methodological requirements are successfully demonstrated before. *Reliability *measures (internal consistency and test-retest stability) were found to be good across countries [9]. Regarding *validity *it was shown that the internal structure of the MRS across countries was sufficiently similar to conclude that the scale really measures the same phenomenon [9].

The comparison with another scale for aging women – although not a validated HRQoL scale (Kupperman) – showed sufficiently good correlations of the total score, which is compatible with the notion of a good criterion-oriented validity. The same is true for the comparison with the generic quality-of-life scale SF36 where also high correlation coefficients have been shown [3-5]. Another fact in favor of the scale is that it was translated into 10 languages so already [7-9].

Having the above-mentioned psychometric data available, a point was reached to critically evaluate the capacity of the scale to reliably measure health-related effects of hormone treatment independent from the severity of complaints and – in addition – to the comparison of treatment effects measured by the MRS scale and the subjective assessment by the treating physician. To this end, many clinicians use the term "validity" and mean high utility for clinical work or research.

The only hormone treatment study with the MRS scale as outcome measure in women during menopausal transition we could get data for methodological analysis was the above described postmarketing study. We hope to repeat/confirm this analysis with data of a more stringently designed clinical trial. But even on the basis of a methodologically weak dataset, in absence of other data, we got re-assuring methodological information about the MRS scale.

It is a well-established experience that women with menopausal complaints respond to hormone therapy with a marked improvement of the HRQoL. This is what the MRS scale should be able to detect.

We saw that the increased mean MRS total score at baseline (before treatment) markedly decreased after 6 months under treatment indicating a significant improvement of complaints & HRQoL. This was also the case for the mean scores of the three subscales. These data cannot disentangle the effect of treatment and "natural variation" of complaints over time. This however was not the point: It was not the intention of this paper to evaluate effectiveness of hormone therapy in an uncontrolled post-marketing study.

The absolute improvement of symptoms during treatment was 9.3 points of the MRS total score on average. This is equivalent to 36% of the baseline score, and similar also for all three subscales. In other words, the MRS scale was shown to be successful in detecting treatment effects. The impressive magnitude of the therapy-related improvement of HRQoL should be obviously discussed in the context of selection of women with complaints susceptible for this kind of treatment by the participating gynaecologists. Another critical remark is that we cannot comment as to what extend the MRS scale is able to measure true or placebo treatment effects. But this is more a question concerning efficacy and the study draw any conclusions in this regard by definition of the study design.

To answer the question whether the sensitivity of the MRS scale is good enough to detect even treatment-related changes in women with only little or mild symptoms as compared with severe ones, the analysis was stratified. An improvement of complaints/QoL was seen in an increasing degree in patients with little, mild, moderate and severe symptoms at baseline. The relative improvement increased with the degree of severity of symptoms at baseline, which is consistent with the general expectation. It seems to be important to underscore: The MRS scale seems to detect also a positive treatment effect in women with little complaints – although to a lesser degree.

Moreover, we showed the capacity of the MRS scale to determine therapeutic efficiency with another approach: a face-value-comparison with norm values of the population [2,3]. The level of complaints in patients before therapy expressed a higher degree of severity (higher MRS total score). After 6 months of hormone treatment the frequency distribution of patients with a certain severity of complaints returned towards a similar distribution as observed in the general population. The extreme proportion of patients with no/ little complaints after therapy should be again seen in the context of apparent patient selection (patients were not only treated because of their symptoms but also for other indications such as prevention) and/or effects of the interaction of patients with the treating physician (who also administered the MRS. Thus, we cannot exclude that such a biases have inflated the impression of a "too positive therapy efficiency". But we do not intent to draw conclusions about therapeutic efficiency anyway. It is another way to look at therapeutic efficiency with the assistance of the MRS scale. Although this indicates at least that comparisons with norm values could be helpful for interpreting results of intervention studies, we are not recommending formal statistical testing of differences between patient groups and the reference values of the population: Patients are usually too different from the general population, a difference hard to adjust for. It is just a visual comparison (as in Table 3) to get a crude idea for the interpretation of results.

The MRS scale was also tested whether it predicts the therapeutic assessment of the treating physician. At face value, the individually assessed efficiency of hormone treatment by the treating gynaecologists was comparable with the assessment by the MRS scale, i.e. using a simple dichotomization of the treatment effect in "*successful*" and "*not successful*" for both the subjective opinion of the physician and the result of the MRS scale: The sensitivity (correct prediction of a positive assessment by the physician) was 70.8% and specificity (correct prediction of a negative assessment by the physician) 73.5%. In other words, the MRS scale fits well with the subjective assessment of the treatment effect estimated by the physician. However, conclusions have to be drawn very carefully because of a possibly inherent bias that may have inflated the positive result: The subjective assessment of "success" by the treating physician was obviously not as independent from the assessment by the MRS scale as desirable because the physician applied the scale to the patient. Even without being able to recall the result of the MRS six month ago or to calculate and compare the total score of both administrations, the interaction with the patients is likely to have introduced this bias in the direction of a higher compatibility between both assessments.

Although the result may too positive compared with a blinded, really independent assessment, it permits to generate the working hypothesis of a sufficiently good prediction of the therapeutic effect by means of the MRS scale. This needs to be confirmed with better data, i.e. data from a blinded, independent comparison, i.e. with the currently used, self-administered MRS scale.

The aim of this exercise was only to demonstrate that the MRS scale may well predict the clinical opinion about efficiency of hormone therapy, what was not empirically shown before. We recommend the MRS as standardized/validated "objective" scale for use in clinical studies, although some aspects discussed above need confirmation in a new study. Moreover, since the scale is already broadly used at the international level, it is important to sensitise users about some lacking information or weak evidence.

The limitations of this study should be shortly summarized. First of all, this study was performed in a dataset where an earlier version of the MRS scale was used, i.e. the scale was not self-administered but completed in an interview of the physician with the patient. This could have influenced the magnitude of the absolute scores of the total and sub-scales. As far as pre-/post-treatment changes are concerned, the magnitude of the absolute changes may have been more influenced than the relative changes of the HRQoL assessment of the patients as discussed in this paper. Another problem along the same line is that we had to transform the old coding system into the new one. This was done with a simple linear transformation and is not likely to have introduced any bias. Another limitation is that this is the first study we are aware of for this kind of assessment of the validity to measure therapeutic intervention.

It is not likely that the main conclusions of the study are materially biased. However, the results should be cautiously used (e.g., for planning clinical trials or outcomes studies) as long as not confirmed with data obtained with the currently used self-administered MRS scale without potential influence of the physician. It can be assumed that a new study with the currently recommended MRS scale – in the sense of "patient-reported-outcome" – would demonstrate positive results but to a lesser degree.

## Conclusions

The MRS scale showed some evidence for its ability to measure treatment effects on quality of life across the full range of severity of complaints in aging women. This however needs confirmation in other and better-designed clinical studies.

## Competing interests

The authors FS and SG are employees of the company that produces HRT products.

We cannot however see any conflict of interest as far as the methodological aspects of the validation of the MRS scale are concerned.

## Authors' contributions

LAJH: responsible for the collection and evaluation of the data, and involved in writing of the paper. DMT: responsible for building the database of this publication, responsible for the statistical evaluation, and contributed to writing of the paper. FS: responsible for the post-marketing study and designing this paper, contributed to the manuscript. SG: responsible for designing and overseeing the post-marketing study of Climen, contributed to writing and revising of the paper JS: responsible for the field work of the post-marketing study, setting up the initial database, and for the preparation of the subset of data used for this publication. HPGS: Major responsibility in developing the MRS scale, contributed to writing/revision of the manuscript.
